# Symptoms of depression are associated with physical inactivity but not modified by gender or the presence of a cardiovascular disease; a cross-sectional study

**DOI:** 10.1186/s12872-019-1065-8

**Published:** 2019-04-25

**Authors:** Retze Achttien, Jan van Lieshout, Michel Wensing, Maria Nijhuis van der Sanden, J. Bart Staal

**Affiliations:** 1 Radboud Institute for Health Sciences, IQ healthcare, Radboud university medical center, Geert Grooteplein 21, 6500 HB Nijmegen, The Netherlands; 2 Health services research and implementation science in healthcare, Heidelberg University, Heidelberg, Germany; 3 Research group Musculoskeletal Rehabilitation, HAN University of Applied Sciences, Nijmegen, The Netherlands

**Keywords:** Physical activity, Symptoms of depression, CVRM

## Abstract

**Background:**

Depressive symptomatology may act as a barrier to enhance physical activity. This phenomenon is predominantly found in patients with an established cardiovascular disease (CVD) and in female patients. This cross-sectional study investigated (1) the association between symptoms of depression and physical inactivity, and (2) whether this association is different between primary and secondary prevention patients, and between men and women.

**Methods:**

The study design concerns a secondary analysis of baseline data from a randomized clinical trial, including primary and secondary prevention patients (*n* = 2184; mean age 71.6 ± 8.94), from 34 general practitioner panels. The Rapid Assessment of Physical Activity questionnaire (RAPA) was used to measure patient reported activity levels. Symptoms of depression were determined using the Patient Health Questionnaire (PHQ-9). Multilevel linear regression analysis was used to explore the association between symptoms of depression and physical activity while adjusting for confounders. Gender and whether or not having a CVD were considered as potential effect modifiers for the association between symptoms of depression and inactivity.

**Results:**

Symptoms of depression were associated with lower levels of physical activity. This association was neither different for men and women nor for primary and secondary prevention patients.

**Conclusion:**

In primary care patients’ symptoms of depression were associated with physical inactivity. This association was not modified by gender or the presence of a CVD. Future research should focus on lifestyle interventions aiming at the increase of physical activity levels, while emphasizing on improving symptoms of depression in men, women, and patients both with and without a history of CVD.

## Background

Regular physical activity reduces the progression of atherosclerosis, and consequently optimizes the cardiovascular risk profile, and thereby decreases the incidence of cardiovascular events [1]. Exercise capacity is mentioned as one of the strongest modifiable predictors of mortality among patients at risk for developing a cardiovascular disease (CVD) [2]. Despite this well-documented effect, exercise activity is limited among many patients at high cardiovascular risk [3, 4]. Stimulating physical activity is therefore a core component of cardiovascular risk management (CVRM) in primary care [5]. However, to tailor CVRM interventions to individual patients’ needs it is critical to identify patient characteristics that are associated with physical inactivity. Patient characteristics such as depressive symptomatology [6–8], female gender [9, 10], and suffering a CVD [11] have been found to deteriorate physical activity levels.

Depressive symptomatology may impede the successful stimulation of physical activity and patients’ efforts to improve lifestyle in CVD [6–8]. On the other hand, symptoms of depression can be reduced by increasing physical activity levels among CVD patients [12–14]. Depressive symptomatology predicts the incidence of CVD, worsens its prognosis, and is mentioned as an independent risk factor for mortality in CVD patients [15–17]. The prevalence of depressive symptomatology increases with age [11, 18], smoking status [18, 19], and is more prevalent in women compared to men [9, 10]. Female gender is, independent from experiencing symptoms of depression, also associated with lower activity levels [20, 21]. This might, for instance, be caused by lower perceived self-efficacy and self-management levels in women [22]. Additionally, women experience a CVD at an older age than men, and consequently suffer comorbidity more frequently, resulting in lower activity levels [23–25]. More attention for female gender during CVRM in clinical practice is therefore advocated in the literature [9, 10].

Symptoms of depression and consequently lower activity levels are also more frequently reported after a cardiovascular event [20, 26, 27]. Research demonstrating this relationship between depression and inactivity is predominantly conducted during or after (cardiac) rehabilitation in patients with an established CVD (secondary prevention patients) [20]. It may be assumed that this relationship also exists in primary prevention patients (patients at high cardiovascular risk without an established CVD), but data to support this hypothesis are lacking. Yet, insight into possible differences in the association between symptoms of depression and physical inactivity among primary and secondary prevention population might be important for tailoring CVRM to these respective populations [28].

To further explore the association between symptoms of depression and physical inactivity we used baseline data from a large clustered randomized clinical trial (RCT) conducted in primary care patients in the Netherlands [28]. The present study aims to investigate (1) the association between symptoms of depression and physical inactivity, and (2) whether this association is different between primary and secondary prevention patients, and between men and women. These analyses should contribute to our understanding about how to tailor patient-specific lifestyle interventions aiming at a healthy and physically active lifestyle, offered during CVRM in primary care.

## Methods

### Study design

The study design concerns a cross-sectional secondary analysis of baseline data from a clustered RCT [28]. The RCT was part of the international Tailored Implementation for Chronic Diseases (TICD) project. This project developed and tested programs to improve healthcare for patients with chronic diseases in five European countries, and examined methods for tailoring such programs to target groups and settings [29]. The Medical Ethics committee Arnhem-Nijmegen, the Netherlands, waived approval (number 2013/229) for the RCT [30]. The declaration of Helsinki was followed. In our study, we followed the STrengthening the Reporting of OBservational studies in Epidemiology (STROBE) statement to report our cross-sectional study [31].

### Setting

A random sample of 1600 general practices in seven geographical areas in the Netherlands was invited to participate in the RCT [28], resulting in a sample of 34 practices and 2184 patients that were included in our study. Baseline data, used in our study, were collected by the general practitioner (GP) or a nurse practitioner in the years 2013 and 2014. GP practices were only invited to participate if a practice assistant was working in the practice with CVRM in the portfolio.

### Participants

All patients were 18 years or older and were able to provide informed consent. Exclusion criteria were diabetes mellitus, pregnancy and lactation, terminal illness, cognitive impairment, and insufficient mastery of the Dutch language hindering reading and answering the questionnaires. We included the baseline data from a RCT [28] that focussed on CVRM for primary and secondary prevention patients. Primary prevention patients had an estimated 10-year risk score of 20% or higher for morbidity and mortality due to CVD. Secondary prevention patients had a cardiovascular event (predominantly coronary artery disease or less frequently a stroke) in history or suffered from a chronic heart disease or peripheral arterial disease. Patient selection was based on the following International Classification of Primary Care (ICPC) codes: K74-K76, K85-K92, K99.1, and T93.

### Variables

The outcome variable was the degree of physical activity measured by the patient reported Rapid Assessment of Physical Activity questionnaire (RAPA) [32]. The predictor variable was symptoms of depression measured by the Patient Health Questionnaire-9 (PHQ-9) [33]. Gender and whether or not suffering a CVD were considered potential effect modifiers for the association between symptoms of depression and inactivity. The experienced degree of control on own health status, measured using the Patient Activation Measure questionnaire (PAM) [34], age, and smoking status were considered as potential confounders.

### Data sources and measurements

Data were collected using structured questionnaires. Questionnaires were sent by mail to all patients and returned by mail as well. Data collection was performed equally in primary and secondary prevention patients.

### Study size

The sample size that was used in the RCT [28] on which we focused was based on a sample size calculation for the cluster randomized trial [30]. The calculation indicated that 450 patients per group (primary or secondary prevention) would be needed for the RCT (15 primary and 15 secondary prevention patients per cluster, sampled in 30 practices). The patient response to participate in the RCT was 41.8% of the invited patients at baseline [28].

### Quantitative variables

All patients filled out the RAPA, PHQ-9, PAM and a smoking questionnaire. The RAPA (9-items) is a valid measure of physical activity for use in clinical practice [32]. RAPA evaluates a wide range of physical activity levels, from sedentary to vigorous activity, as well as strength and flexibility training [35]. Each question has a ‘Yes’ or ‘No’ option. The score ranges from 1 to 7; a score of 6–7 points is considered “active”, 4–5 points as “suboptimal active”, and ≤ 3 points is defined as “sedentary” to “under-active regular-light activities”. Missing values were only considered as such if no questions were filled in. In the data-analysis, we used the RAPA as a continuous score.

The PHQ-9 is a validated depression module for primary care, which scores each of the nine Diagnostic and Statistical Manual of Mental Disorders (DSM-IV) criteria as “0” (not at all) to “3” (nearly every day), with a total score ranging from 0 to 27 points [32]. The total score can also be categorized in 5 severity levels; 0–4 none, 5–9 mild, 10–14 moderate, 15–19 moderately severe, and 20–27 severe symptoms of depression. The total score on the PHQ-9 was used as a continuous value in the statistical analysis.

The PAM is a valid and reliable instrument assessing patients’ activation measure [34]. The PAM survey measures patients on a 13–52 scale, revealing insight into an array of health-related characteristics (attitudes, motivators, behaviors, and outcomes). In case 1 or 2 separate questions were not answered (missing), the total PAM score was subsequently divided by 12 or 13 and multiplied by 13. The PAM was excluded from analysis if fewer than 11 out of 13 questions were answered. This strategy was in accordance with the guideline for interpretation of the questionnaire [34].

The smoking status was derived from the first question of a standardized questionnaire on smoking habits [36]. There were 4 possible answers options: “Yes I smoke”, “No I quite last 6 months”, “No I quite more than 6 months ago”, or “No I never smoked”. We dichotomized this into Yes or No in the analysis. The covariate age was used as a continuous variable in the analysis.

### Statistical methods

Data analysis was performed using SPSS (version 23, IBM Corp.) Data were described using means, standard deviations, numbers, and percentages. Patient characteristics and the outcome of (completed) questionnaires were presented for the total study population. Outcomes were presented on a dichotomous or continuous scale.

First, independent sample T-tests and/ or Chi-square tests were used to compare the outcome variable, the predictor, and potential confounders between primary and secondary prevention patients, and between male and female patients. Second, the assumptions for multilevel linear regression analyses (i.e. linear relationship between the independent and dependent variables, multivariate normality, no or little multicollinearity, and homoscedasticity) were checked [37]. Then, multilevel linear regression analysis (95% confidence intervals (CIs)), using random slopes and intercepts, was performed to determine the association between symptoms of depression and physical inactivity. We adjusted for the potential confounders; score on the PAM questionnaire, age and smoking, gender and whether or not suffering a CVD. Third, we explored if the association between symptoms of depression and physical inactivity was different between primary and secondary prevention patients, and between men and women, by adding the interaction terms (primary/secondary prevention group*PHQ-9 and gender*PHQ-9 respectively) to separate models. A *p* value < 0.05 was considered as statistically significant.

## Results

### Participants and descriptive data

Table 1 presents the descriptive statistics and responses to the questionnaires.

**Table 1:** Descriptive characteristics of the study population

	Patient group					
	Primary prevention patients			Secondary prevention patients		
	Female (*n* = 407)	Male (*n* = 843)	Total (*n* = 1250)	Female (*n* = 354)	Male (*n* = 580)	Total (*n* = 934)
Age in years, mean, SD	75.74 ± 7.10	73.15 ± 6.79	73.99 ± 6.99	68.83 ± 13.11	68.31 ± 10.80	68.50 ± 11.73
RAPA, mean, SD	4.4 ± 1.62	5.01 ± 1.54	4.82 ± 1.59	4.61 ± 1.55	5.02 ± 1.58	4.87 ± 1.58
Active (RAPA 6–7 points), *n*, (%)	151 (37.1%)	430 (51.0%)	581(46.5%)	137 (38.7%)	299 (51.6%)	436 (46.7%)
Suboptimal (RAPA 4–5 points), *n*, (%)	97 (23.8%)	205 (24.3%)	302 (24.2%)	97 (27.4%)	132 (22.8%)	229 (24.5%)
Underactive (RAPA≤3 points), *n*, (%)	129 (31.8%)	172 (20.4%)	301 (24.1%)	98 (27.7%)	127 (21.9%)	225 (24.1%)
*Missing, n, (%)*	*30 (7.4%)*	*36 (4.3%)*	*66 (5.3%)*	*22 (6.2%)*	*22 (3.8%)*	*44 (4.7%)*
PHQ-9, mean, SD	2.67 ± 3.35	1.78 ± 3.08	2.05 ± 3.19	4.23 ± 4.48	2.57 ± 3.49	3.18 ± 3.96
No symptoms of depression (0–4), *n*, (%)	298 (73.2%)	719 (85.3%)	1017 (81.4%)	208 (58.8%)	445 (76.7%)	653 (69.9%)
Mild symptoms of depression (5–9), *n*, (%)	64 (15.7%)	77 (9.1%)	141 (11.3%)	93 (26.3%)	84 (14.5%)	177 (19.0%)
Moderate symptoms of depression (10–14), *n*, (%)	13 (3.2%)	22 (2.6%)	35 (2.8%)	26 (7.3%)	25 (4.3%)	51 (5.5%)
Moderately severe symptoms of depression (15–19), *n*, (%)	7 (1.7%)	3 (0.4%)	10 (0.8%)	10 (2.8%)	4 (0.7%)	14 (1.5%)
Severe symptoms of depression (20–27), *n*, (%)	0	4 (0.5%)	4 (0.3%)	4 (1.1%)	2 (0.3%)	6 (0.6%)
*Missing, n, (%)*	*25 (6.1%)*	*18 (2.1%)*	*43 (3.4%)*	*13 (3.7%)*	*20 (3.4%)*	*33 (3.5%)*
PAM, mean, SD	35.31 ± 8.47	36.70 ± 8.55	36.27 ± 8.5	36.41 ± 7.59	38.45 ± 7.56	37.68 ± 7.63
*Missing, n, (%)*	28 (7.4%)	12 (1.4%)	40 (3.3%)	14 (4.1%)	14 (2.5%)	28 (3.1%)
Smoking status						
YES, *n*, (%)	23 (5.9%)	87 (10.5%)	91.2 (8.8%)	52 (15.2%)	77 (13.6%)	129 (13.8%)
NO, *n*, (%)	366 (94.1%)	471 (89.5%)	1107 (89.5%)	291 (84.8%)	490 (86.4%)	781 (86.2%)
*Missing, n, (%)*	*18 (4.4%)*	*15 (1.8%)*	*33 (2.7%)*	*11 (3.1%)*	*13 (2.2%)*	*24 (2.5%)*

*RAPA* Rapid Assessment of Physical Activity Questionnaire, *PHQ-9* Patient Health Questionnaire-9, *PAM* Patient Activation Measure questionnaire

In total, 2184 patients were included in our study (1250 primary and 934 secondary prevention patients), consisting of 761 female patients and 1423 male patient. The high-risk population consisted of 33% (mean age 75.7 ± 7.0) female patients and in the CVD population 38% (mean age 68.8 ± 13.1). Primary prevention patients were significantly older than secondary prevention patients (*p* < 0.05; 73.99 ± 6.99 vs 68.50 ± 11.73). In total, 110 (5%) patients filled out the RAPA inadequately. For the PHQ-9, PAM and smoking status, the non-response was as follows: 76 (3,4%), 68 (3,1%), and 57 (2,6%).

### Outcome data

Female patients in the primary prevention group scored on average 4.40 (±1.62) points on the RAPA and in the secondary prevention group 4.61 (±1.55) points. This difference is statistically significant (*p* < 0.05). Male patients were in both groups physically more active (*p* < 0.01) compared to female patients (primary prevention group 5.01 ± 1.54 vs 4.4 ± 1.62 and secondary prevention group 5.02 ± 1.58 vs 4.61 ± 1.55, *P* < 0.01). In the primary and secondary prevention group, about 50% of the patients scored “active” on the RAPA (6–7 points), 25% scored “less than suboptimal active” (4–5 points), and 25% scored “sedentary” to “under-active regular-light activities” (1–3 points).

Most patients in this study did not report symptoms of depression (81.4% in the primary prevention group and 69.9% in the secondary prevention group). Mild and moderate symptoms of depression were more frequently reported in the secondary prevention group compared to the primary prevention group (mild symptoms of depression; 11,3% vs 19.0% and moderate symptoms of depression 2,8% vs 5.5%, *p* < 0.01). Reported symptoms of depression were significantly higher in patients suffering a CVD (women: 4.23 ± 4.48, men 2.57 ± 3.48, *p* < 0.01) compared to primary prevention patients (women: 2.67 ± 3.35, men 1.78 ± 3.08, *p* < 0.01).

In the secondary prevention group, both female and male patients were more often reported to be a smoker compared to the primary prevention group. The primary prevention group reported lower scores on the PAM compared to secondary prevention patients (36.27 ± 8.55 vs 37.68 ± 7.63, *p* < 0.01). There was no difference in PAM score between women and men.

### Main results

Assumptions for multilevel linear regression analyses were not violated and no effect for clustering was found (ICC = 0.02). Table 2 presents the multilevel linear regression analyses concerning the association between symptoms of depression and physical inactivity (using the RAPA as a continuous variable), adjusted for confounders. There was a significant association (*p* < 0.01) between symptoms of depression and physical inactivity, indicating that a 1-point increase on the PHQ-9 questionnaire is associated with a decrease of 0.06 on the RAPA. The association between symptoms of depression and physical inactivity was neither modified by the interaction term primary/secondary prevention group*PHQ-9 (*p* = 0.47: Table 2, model 2) nor by the interaction term gender*PHQ-9 (*p* = 0.44: Table 2, model 3).

**Table 2:** Results of multilevel linear regression analyses presenting the association between symptoms of depression and physical activity

	Model 1 95% C.I.				Model 2 95% C.I.				Model 3 95% C.I.			
RAPA (1–7)	B	Lower	Upper	*P*	B	Lower	Upper	*P*	B	Lower	Upper	*P*
PHQ-9 (0–27)	−0.06	−0.08	−0.04	< 0.01	−0.08	−0.14	−0.02	0.01	−0.09	−0.15	−0.02	0.01
Gender (M/ F)	0.33	0.18	0.48	< 0.01	0.33	0.18	0.48	< 0.01	0.29	0.10	0.47	< 0.01
Patient group (SP/PP)	−0.01	− 0.15	0.14	0.92	−0.05	− 0.22	0.12	0.55	−0.02	− 0.16	0.12	0.82
Age (Y)	−0.03	−0.03	− 0.02	< 0.01	− 0.03	−0.04	− 0.02	< 0.01	− 0.03	−0.04	− 0.02	< 0.01
Smoking status (Y/ N)	0.35	0.13	0.57	< 0.01	0.35	0.13	0.56	< 0.01	0.34	0.13	0.56	< 0.01
PAM (0–13)	0.02	0.01	0.03	< 0.01	0.02	0.01	0.03	< 0.01	0.02	0.01	0.03	< 0.01
SP/PP group*PHQ-9					0.01	−0.02	0.05	0.47				
Gender*PHQ-9									0.02	−0.02	0.05	0.44

* means SP/ PP group multiplied by PHQ-9

Rapid Assessment of Physical Activity Questionnaire. *PHQ9* Patient Health Questionnaire 9, *PAM* Patient Activation Measure questionnaire score, *SP* secondary prevention patients, *PP* primary prevention patients

## Discussion

### Key results

The aim of this study was to investigate (1) the association between symptoms of depression and physical inactivity, and (2) if this association is different between primary and secondary prevention patients, and between men and women. Our results revealed that depression was associated with physical inactivity in both primary and secondary prevention patients in primary care. However, this association was not modified by gender or the presence of a CVD.

### Strengths and limitations

Patient baseline data (from the RCT [28]) were collected from GP databases, considering an even distribution of primary and secondary prevention patients. However, selection bias may have influenced the outcome in our study. In total 34 GP facilities participated in the RCT, while 1600 practices were invited to participate [28]. In the practices, a nurse practitioner had to be employed who had CVRM in the portfolio. Therefore, it is possible that predominantly data from higher quality GP practices were used in our study.

The selection criteria for patients being included in our study were standardized, using ICPC diagnose codes (mostly objective criteria). Selection bias may have occurred in the RCT by a low patient response (41.8% at baseline) to the invitation to participate [28]. It could be, for example, that patients with no or minor depression predominately participated in our study. However, this selection bias was probably the same in primary and secondary prevention group. Other factors (covariates) associated with physical activity levels and/ or symptoms of depression, for which we did not adjust in the analysis, may have been different between the primary and secondary prevention group. Factors such as personal (scheduling, motivation), social (family support) and environmental (cost, access) barriers and biological correlates (body mass index, physical co-morbidities) may have acted as confounders [6, 20, 24, 38]. In particular, the body mass index might have been a potential confounder, as it has been reported to be associated with lower activity levels in depressed patients [39]. We did not correct for the BMI variable in our model, due to the substantial number of missing data (77%). This is a limitation of our study.

Validated questionnaires were used in our study. Only a limited number of patients did not fill out the questionnaires correctly. We consider this as a strength of our study. Due to the cross-sectional study design, however, it is not possible to draw any causal relationship. This is an important limitation of this study design.

### Interpretation

We revealed the association between symptoms of depression and physical inactivity in a primary care population consisting of primary and secondary prevention patients. In addition, to our knowledge, this is the first study that investigates the possible difference in the association between depression and inactivity between primary and secondary prevention patients, and between men and women.

Increased symptoms of depression and consequently physical inactivity were previously reported in secondary prevention patients during or after cardiac rehabilitation [11, 25]. In addition, it was reported that adults with major depression disorders do have low levels of physical activity [39]. Moreover, in secondary prevention patients it has also been found that inactive patients are more often depressed and that the more patients get depressed, the less active they become [12, 13]. This is in line with our findings in secondary prevention patients, and we also found that this association exists in primary prevention patients. It should be noted that our population predominantly had no (81.4% of the primary and 69.9% of the secondary prevention patients) or only minor symptoms of depression.

The association between depression and physical inactivity was not always consistently reported. In a narrative review [20] for example, authors concluded that during cardiac rehabilitation, depression was associated with physical inactivity in 63% of the studies included. This association was lower (57% of the studies included) in secondary prevention patients who did not attend a rehabilitation program [20]. Differences in findings might be due to the level of symptoms of depression that patients suffer and the degree of patients’ inactivity. Patients included in our study had relatively low levels of symptoms of depression compared to other studies [20]. In the secondary prevention group, this could be the result of multidisciplinary (cardiac) rehabilitation that patients may have followed prior to entering our study, which aims for improving symptoms of depression.

Overall, our study population was also less active compared to patients included in other research [20, 40]. This could be explained by the fact that we included older patients, who might suffer more comorbidity and are therefore less active [23–25]. On the other hand, aging is reported to be inconsistently associated with lower activity levels and exercise adherence among secondary prevention patients [20]. Aging is, however, related to higher prevalence’s of symptoms of depression [11, 18]. Attending cardiac rehabilitation in the past seems not to affect activity levels in the long term and might therefore not affect our findings [4].

We reported higher activity levels in men compared to women in both the primary and secondary prevention group. This has also been reported in earlier research [9, 10, 23, 24]. In our study, men and women scored equally on the PAM, while in previous research it was shown that women had lower self-efficacy than men [22]. In our study we found lower scores on the PAM in the primary prevention group compared to the secondary prevention group. A positive impact of higher self-regulation and self-efficacy, proper health status, positive intention, internal locus of control, and absence of negative illness beliefs is, for example, are consistently related to exercise maintenance in CVD population [20, 41] and the non-disease population [25]. Therefore, we treated the PAM as a potential confounder in the analysis. And finally, in the secondary prevention group were more smokers than in the primary prevention group. Smoking has previously been reported as inconsistently related to inactivity among CVD patients [20, 42], but is associated with increased symptoms of depression levels [19]. Smoking was therefore also treated as a potential confounder in the analysis.

### Generalizability

Dutch citizens are registered by a GP. The GP has a medical record of almost all patients, and therefore every patient who is eligible for CVRM within the participating practices is in the original selection from which the sample has been taken. Also, the distribution of one third female and two third male patients is representative of the CVRM population in the western primary healthcare facilities. Only 3.8% of the patients had no Dutch nationality, and therefore generalizability to people originating from non-western countries could be limited.

## Conclusion

Symptoms of depression are associated with physical inactivity. This association was not modified by gender and the presence of a CVD. Future research should focus on lifestyle interventions aiming at the increase of physical activity levels while simultaneously improving symptoms of depression, despite gender difference and the presence of a CVD. Longitudinal research should be performed to re-confirm the present findings.

## Abbreviations

CVD: Cardiovascular disease
CVRM: Cardiovascular risk management
DSM-IV: Diagnostic and statistical manual of mental disorders
GP: General practitioner
ICPC: International classification of primary care
PAM: Patient activation measure questionnaire
PHQ-9: Patient health questionnaire
RAPA: Rapid assessment of physical activity questionnaire
RCT: Clustered randomized clinical trial
STROBE: STrengthening the reporting of OBservational studies in epidemiology
TICD: Tailored implementation for chronic diseases
